# Determination of an optimum sampling unit of *Ricania shantungensis* (Hemiptera: Ricaniidae) eggs in persimmons

**DOI:** 10.1371/journal.pone.0265083

**Published:** 2022-03-09

**Authors:** Sunghoon Baek, Hwa-Young Seo, Chang-Gyu Park

**Affiliations:** 1 Department of Industrial Entomology, Korea National College of Agriculture and Fisheries, Jeonju, Republic of Korea; 2 Chungnam Agricultural Research and Extension Services, Yesan, Republic of Korea; US Department of Agriculture, UNITED STATES

## Abstract

Even though the optimum sampling methods for invasive pests are very important in newly invaded areas, the standard sampling unit of *Ricania shantungensis* is still undeveloped in persimmons. Among all developmental stages of *R*. *shantungensis*, the egg has close relationship between its density and subsequent tree damage. Thus, this study was conducted to suggest an optimum sampling unit for *R*. *shantungensis* eggs in persimmons based on characteristics of its within-tree distribution pattern. The within-tree distribution pattern was characterized with 60 persimmon trees by cutting 12 branches at three vertical levels (low, middle, and high) in four horizontal criteria (east, west, south, and north) per tree. The sampling units were determined based on coefficient of variation (CV) and coefficient of determination (*r*^2^) calculated from egg mass numbers per 10 cm from the tip within a branch. In numbers of *R*. *shantungensis* egg masses, there was no significant difference (*P* > 0.05) horizontally, but significant (*P* < 0.05) difference vertically. More *R*. *shantungensis* eggs were found on terminal branches of each trunk. The 60 cm from the tip of branches in the terminal positions of each trunk was selected as the optimum sampling unit for *R*. *shantungensis* in persimmons because this unit has the lowest CV value and more than 0.9 of *r*^2^ value. Even though the optimum sample number per tree should be determined field-specifically, it would be acceptable to sample two or three branches by considering this pests’ recognizable damage level. This small sampling unit could make the sampling of *R*. *shantungensis* become more economical, precise, and consistent in persimmon fields.

## Introduction

After the first report in 2010 of outbreak for an invasive species, *Ricania shantungensis* (Hemiptera: Ricaniidae), this pest became an economically important pest in Korea [1]. In agriculture, serious economic damage has been reported mainly in fruits trees because *R*. *shantungensis* can lay eggs only in arboreal plants and its oviposition mainly causes damage by blocking the currency of plant saps in newly developed branches [2]. This pest is known to have host plants of 138 species in 62 families including economically important fruits crops such as persimmon, chestnut, peach, apple and so on [3]. This broad host range with limited natural enemies might allow *R*. *shantungensis* to quickly establish populations in new areas [1]. According to the paper [1], its spread would continue to almost all areas of Korea. Until now, *R*. *shantungensis* has caused the most serious economic damage to apples and chestnuts, among all host plants in Korea [4]. However, there is a high possibility that persimmons become one of the most damaged crops in the near future. There are multiple reasons. First, a persimmon is one of the most preferred hosts for *R*. *shantungensis* adults among whole fruits trees [3]. Second, its field locations are generally located within or near forests in which there are diverse alternative hosts for this pest [4]. Finally, the biggest persimmon cultivation areas in Korea are already neighboring the outbreak areas of this pest [1].

In Korea, chestnuts are spread out evenly without aggregated cropping areas. However, approximately 67% of persimmon production in Korea is in a certain area, Kyeongsangnam-Do [5], in which economic damage of *R*. *shantungensis* has not been reported yet [1]. Therefore, regular and continuous monitoring for *R*. *shantungensis* is required in these areas to check whether this pest occurs or not, to delineate contaminated areas, and to determine this pest’s density. Otherwise, outbreak of *R*. *shantungensis* could occur in persimmon producing areas in the near future. For efficient sampling of *R*. *shantungensis*, a sampling unit for persimmon should be determined. This sampling unit not only determines the nature of a population but also affect the components of sampling plans such as sampling techniques, sampling number, spatial pattern of samples, and timing of sampling [6–9]. In general, sampling units are determined based on a few criteria including suitable size, consistency, the balance between cost (e.g., labor and time) and variation (e.g., precision), stability, possibility of delineation in the field, and so on [9, 10].

In sampling of *R*. *shantungensis*, the target developmental stage is egg because samplings for its nymphs and adults are very difficult [9]. In chestnut fields, the 50 cm branch tip was suggested as an optimal sampling unit for *R*. *shantungensis* [9]. This study [9] also showed that the within-tree distribution of *R*. *shantungensis* eggs were not different vertically and horizontally. However, Kim et al. [11] found the most *R*. *shantungensis* eggs on upper crown within a blueberry tree. This discrepancy indicates that within-distribution of *R*. *shantungensis* eggs would be different on persimmons compared to chestnuts. Until now, there is no report for within-distribution and sampling unit of *R*. *shantungensis* in persimmons.

Therefore, the goals of this study were (1) to find out the characteristics of within-tree distribution of *R*. *shantungensis* eggs, (2) to suggest an optimum sampling unit, and (3) to develop a model to estimate numbers of *R*. *shantungensis* eggs on a whole branch with samplings on its partial parts in persimmon trees.

## Materials & methods

### Study site and sampling

This study was conducted in two persimmon fields in Jeongdong-Myeon, Sacheon-Si, Kyeongsangnam-Do, Korea in 20201. Each farm owner allowed this experiment. In both fields, *R*. *shantungensis* was first reported in 2018 [1]. One persimmon field (N 35.057406, W 128.094517) was a managed commercial field. In this field, the height of persimmon trees was strictly controlled. Diverged trunks from a main trunk were manipulated to grow almost horizontally. Most branches within a tree were reachable to hands without any other tools. However, the other field (N 35.057915, W 128.092055) was abandoned for the last few years. Even though a main trunk was diverged, the diverged trunk grew diagonally. Almost all branches on terminal trunks required ladders to reach.

In each persimmon field, a total of 30 trees were randomly selected and observed on 25 February, 2021. The 12 whole branches per tree were randomly selected within a tree at three vertical levels (low, middle, and high) in four horizontal criteria (east, west, south, and north). However, it is general to diverge a main trunk into four trunks in persimmon productions of Korea in order to control tree height. Thus, the three vertical levels can be interpreted as terminal, middle, and basal parts of each cardinal direction trunk. Selected branches were cut by using hand pruners (P-300, Hwashin Metal IND Co.; Daegu, Korea) and a pole pruner (1000-DXA, Kamaki Korea; Paju, Korea). Egg masses on branches per 10 cm from the tip of branch were counted and recorded by placing the cut branches on a self-made paper arena (1 m × 2 m) with grids (10 cm × 10 cm). The length of whole branches was also measured and recorded.

### Within-tree distribution of *R*. *shantungensis* eggs

Numbers of *R*. *shantungensis* egg masses on branches were analyzed horizontally and vertically with ANOVA [12]. Statistical analyses were done by combing data of both fields because there was no statistical difference in egg mass densities between two sites (*t* = 1.304; *df* = 58; *P* = 0.0153).

### Optimum sampling unit

An optimum sampling unit for *R*. *shantungensis* eggs in a persimmon tree was determined in two criteria as the previous study [9]. First, coefficient of variation (CV) of *R*. *shantungensis* egg mass numbers was calculated among distances (i.e., 10 cm, 20 cm, 30 cm, ⋯) from the branch tip by dividing its standard deviation by its mean. The sampling unit with the lowest CV value has two important ecological meanings in sampling: First, this sampling unit has a relatively higher possibility to find target insects rather than other sampling units with similar variance [9, 13]. Second, the variances of samples become smaller compared to sampling units with similar means [9, 13].

Second, coefficient of determinations (*r*^2^) was estimated from the linear relationships between egg mass numbers on selected sampling units and its whole branch to evaluate its prediction ability of egg numbers on its whole branch. In this study, the *r*^2^ value of 0.9 was used as the minimum criteria. The *r*^2^ value of 0.9 means that the egg numbers on selected sampling units in a whole branch would explain 90% of variances in numbers on its whole branches.

Combined data of both fields were used to decide an optimum sampling unit because there was no statistical difference in egg mass densities between the two sites (*t* = 1.304; *df* = 58; *P* = 0.0153).

### Required sample number

A required sample number is the smallest number of sampling units to satisfy the objectives of the sampling program and achieve the desired precision of estimates [6, 9]. There are multiple equations to calculate a required sample number. Among them, this study used Reusink’s equation [9, 14] because it is difficult to define the degree of the spatial distribution of *R*. *shantungensis* eggs within a tree. The equation is following:

$$N=1/(D^{2}\times m),$$ (1)

where *N* is required sample number, *D* is degree of precision which is the ratio of mean to standard error, and *m* is the mean counts of *R*. *shantungensis* egg masses per selected sampling unit.

### Prediction model of *R*. *shantungensis* egg masses within a branch

The Weibull equation was used to fit cumulative proportion of *R*. *shantungensis* egg mass numbers according to the distances from the branch tip within a branch. The equation is:

$$f(x)=100[1-exp\{-\left({x-a\}}^{b}\right\}\},$$ (2)

where *f*(x) is the cumulative proportion (%) of egg mass counts, *x* is the distance from the tip, and *a* and *b* are parameters.

The cumulative proportion of egg mass numbers per 10 cm were constructed from the frequency data of egg mass counts on sampling locations from the tip to whole numbers within a branch. The model parameters were estimated by using PROC NLIN [12].

## Results

### Within-tree distribution of *R*. *shantungensis*

Number of *R*. *shantungensis* egg masses was significantly different vertically (*F =* 4.757; *df* = 2, 177; *P* = 0.010), but not different horizontally (*F =* 0.367; *df* = 3, 236; *P* = 0.777) (Table 1). More eggs were found on branches located at the terminal parts of each trunk regardless of horizontal directions (Table 1). Thus, the branches of terminal parts of each trunk were selected further analyses.

**Table 1:** Densities of *R*. *shantungensis* egg masses (mean ± SE) according to horizontal and vertical locations of a whole branch within a persimmon tree.

Horizontal direction	Density	Vertical location	Density
East	2.8 ± 0.49 a^1^	High	5.0 ± 0.61 a
West	2.8 ± 0.47 a	Middle	3.1 ± 0.49 b
South	2.4 ± 0.39 a	Low	3.0 ± 0.41 b
North	3.1 ± 0.47 a^1^		

^1^ Means within a column followed by the same letter are not significantly different (*P* > 0.05; Tukey’s HSD test).

### Optimum sampling unit and required sample number

As the size of sampling unit increased, *r*^2^ values also increased (Table 2). However, the CV values were the lowest at 60 cm branch tip among all available sampling units for the branches of terminal parts of each trunk (Table 2). Therefore, 60 cm branch tip of terminal branches within a persimmon tree was suggested an optimum sampling unit for *R*. *shantungensis* eggs.

**Table 2:** Coefficient of variance (CV) for counts of *R*. *shantungensis* egg masses according to selected sampling locations from the tip within terminal branches and coefficient of determination (*r*^2^) of the linear regressions of egg mass counts between on selected sample locations within a terminal branch and on its whole branch.

Sampling unit	10 cm	20 cm	30 cm	40 cm	50cm	60cm	70 cm	~	Whole branch
CV	1.08	1.01	0.92	0.91	0.93	0.85	1.09	~	0.94
*r* ^2^	0.67	0.89	0.96	0.96	0.99	0.99	0.99	~	1

**Fig 1:**
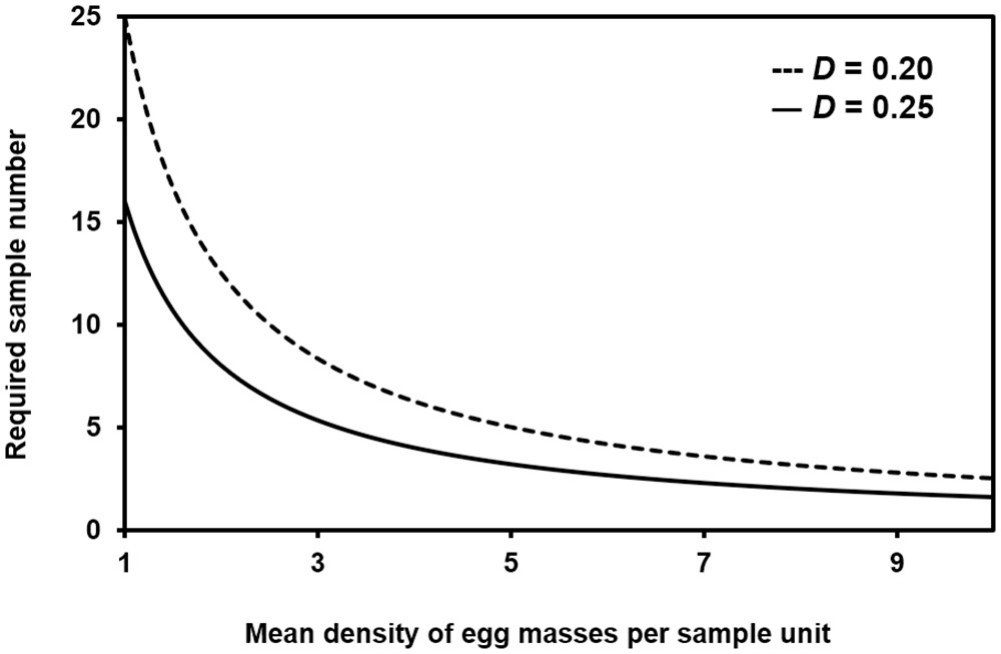
Required sample numbers according to mean densities of *R*. *shantungensis* egg masses in fixed precision level (*D*) of 0.20 and 0.25.

At the precision level of 0.20, the suggested sample number per tree were 25, 13, 9, 7 and 5 at 1, 2, 3, 4, and 5 mean egg mass numbers on a selected sampling unit (60 cm branch tip), respectively (Fig 1). However, the required sample numbers decreased as 16, 8, 6, 4, and 4 at 1, 2, 3, 4, and 5 egg numbers on the sampling unit when the precision level increased from 0.20 to 0.25 (Fig 1). To meet the desired precision level (i.e., *D* = 0.25) of management purposes, the minimum sampling number was 2.7 branches per tree in the studied fields because the mean egg density was 5.9 on the selected sampling unit. For research purposes (i.e., *D* = 0.20), the sample number was 4.2 branches in this study.

### Prediction model of *R*. *shantungensis* egg masses within a branch

**Fig 2:**
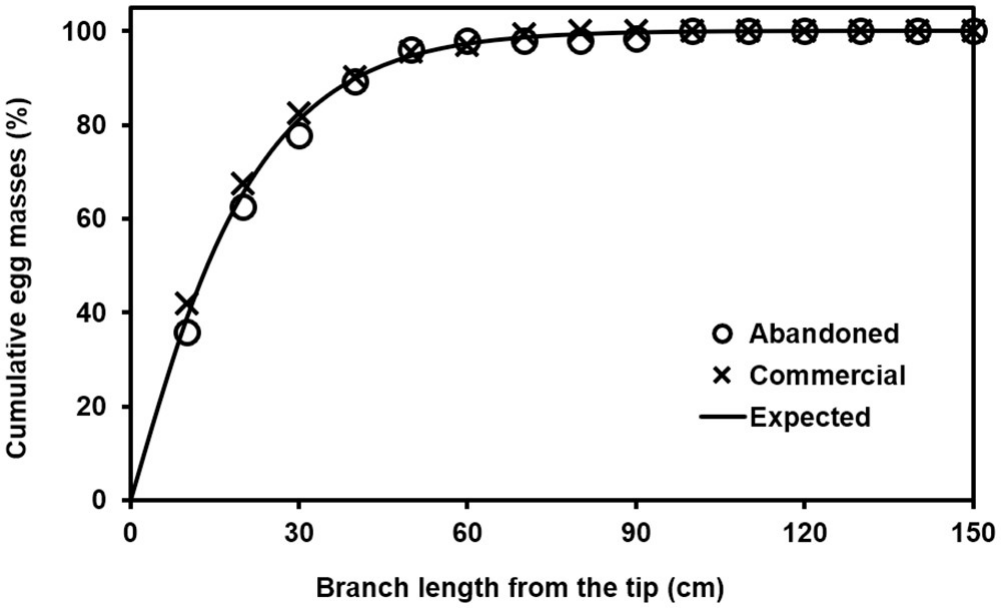
Cumulative egg mass proportion (%) of *R*. *shantungensis* according to distances from the tip within a branch.

The Weibull function could well predict cumulative proportion of *R*. *shantungensis* egg mass counts according to distances from the branch tip (Fig 2, *F* = 399138; *df* = 1, 14; *P* < 0.0001; *r*^2^ = 0.99). In this model, the parameters (estimate ± SEM) were 18.9660 ± 0.1121 and 1.1108 ± 0.0116 for *a* and *b*, respectively. The 14 cm from its tip within a branch could contain roughly 50% eggs on its whole branch, and 76 cm have 99% eggs (Fig 2). Roughly 97% egg masses could be found at the selected sampling unit (i.e., 60 cm branch) in this study (Fig 2).

## Discussion

There was no significant (*P* > 0.05) difference in the number of *R*. *shantungensis* egg masses horizontally within a persimmon tree, but significantly (*P* < 0.05) more eggs were found on terminal branches than basal and middle ones. This result indicates that any living terminal branches regardless of horizontal directions within a tree would be suitable for sampling of *R*. *shantungensis* eggs in persimmon fields. Within a terminal branch, the CV value was the lowest and the *r*^2^ value was 0.99 at 60 cm branch tip. Thus, 60 cm from the tip of branches in the terminal positions of each trunk within a tree should be used as the sampling unit for *R*. *shantungensis* eggs in persimmon fields.

There were conflicting results [9, 11] for within-tree distribution of *R*. *shantungensis* eggs, especially for vertical directions. Baek et al. [9] reported there was no vertical difference in egg numbers within a chestnut tree. They described newly developing branches would evenly distributed regardless of vertical locations by the pruning which makes all remained branches are exposed to enough sunlight in chestnuts. Moreover, *R*. *shantungensis* adults could approach all vertical branches. However, Kim et al. [11] found that *R*. *shantungensis* eggs were located more frequently in upper crowns of blueberries. The reasons for this phenomenon were new branches were mainly developed in upper positions in blueberries and adult could better approach upper locations rather than middle or lower ones. The results of both studies [9, 11] could be explained by an ecological characteristic of *R*. *shantungensis* that its adults have the high ovipositional preference for newly developed branches [9, 15, 16]. In this study, *R*. *shantungensis* adults showed preference on terminal branches of each artificially diverged trunk. The reasons of this ovipositional preference were that new branches were mainly developed from the terminal parts of trunks and flying adults were difficult to approach basal parts of trunks due to thickly developed branches and fruits at terminal parts of trunks. These ovipositional ecologies have been also reported in a different hemipteran species, *Adelges tsuge* [17–23]. In these studies, oviposition of *A*. *tsugae* was more related with amount of newly developed branches compared to absolute branch locations within a hemlock tree because female adults decide their oviposition with secondary metabolites (e.g., amounts of monoterpenes) of trees. Thus, there is a high possibility that *Ricania* species also use chemical materials to find new branches for oviposition like *A*. *tsugae*. Recently, it was also known that one of *Ricania* species, *Ricania speculum*, could use visual cue to find new branches. Adults of *R*. *speculum* were attracted to the green colored sticky trap (closed to newly developed branch color) rather than other colored ones during ovipositional periods [24]. These results indicate that *R*. *shantungensis* female adults might prefer terminal branches of each trunk in height-controlled trees such as persimmon, apple, peach and so on. Whereas, more eggs might be found in upper crowns in naturally grown broad-leaf trees in forests.

The CV values are useful to suggest optimum sampling units for insects with high variances in densities by granting sampling stability and consistency [9, 13, 23]. The high *r*^2^ values between a sampling unit and its whole part could ensure accuracy and representability of sampling. Thus, the optimal sampling unit should be determined as a branch size with having the lowest CV and highest *r*^2^ values as possible. In this study, 60 cm from its tip with a branch was suggested as a suitable sampling unit for *R*. *shantungensis* in persimmon fields because this sampling unit had the lowest CV value and the highest *r*^2^ value among all available sampling units. This sampling unit could be consistent with the criteria [9, 10] of good sampling units.

The sampling unit affects not only sampling but also damage analysis and management [6, 9]. In these aspects, a target developmental stage should be carefully selected. Although *R*. *shantungensis* of all developmental stages (i.e., egg, nymph, and adult) causes damage to persimmon, the main damage by this pest is caused by its oviposition. Female adults scratch branches with their legs, and then lay eggs inside the scratched cracks [16, 25]. These scratches are easily contaminated with plant pathogens and laid eggs interrupt the movement of plants’ nutrients and water [16, 25]. Therefore, the sampling for eggs would increase the possibilities to find the relationship between its density and tree damage rather than nymphs and adults in *R*. *shantungensis*. Moreover, sampling itself for its nymphal and adult stages is very difficult because both nymphs and adults are very active [16, 25], whereas its eggs are sessile [16], easily noticeable due to their distinctive white wool coverings and large size [2], and exposed for a relatively long time, approximately nine months [25]. Finally, it is difficult to manage *R*. *shantungensis* nymphs and adults because they actively move and feed on both arboreal and herbaceous plants [4].

This study showed decreasing required sample numbers as the density of *R*. *shantungensis* increased. The level of economic damage, the economic threshold, and the appropriate response to *R*. *shantungensis* invasions have yet to be determined. More sampling could always increase accuracy and precision of sampling, but sampling efficiency should be considered [6, 9]. In cases of the densities under the economic threshold, precise sampling becomes less important because management is not needed. Thus, more precise sampling is demanded around the economic threshold than the density over and under the economic threshold. In the managed chestnut fields, the average number of *R*. *shantungensis* egg masses at the suggested sampling unit was approximately seven [9]. In this study, the average number was about six. In both fields, it is difficult to find the *R*. *shantungensis* symptoms in dead branches. Therefore, there is a high possibility that the economic threshold of *R*. *shantungensis* is over seven. Even though it is still recommended to decide its sample number to calculate field-specifically by observing egg mass numbers on a few branches, it might be acceptable to sample two or three branches per tree regardless of tree species for management and research purposes, respectively.

The developed model in this study accurately predicted number of *R*. *shantungensis* egg masses within a branch by explain 99% variation of data. This model could be used to estimate egg numbers on a whole branch with just partial sampling of the branch. Thus, it is possible to use smaller sampling units than the suggest one in this study according to the field and crop situations. Moreover, this model would allow interpreting experimental results using different sampling units.

## Supporting information

S1 File Full data of this experiment. (XLSX)
